# PRAS40 promotes NF-κB transcriptional activity through association with p65

**DOI:** 10.1038/oncsis.2017.80

**Published:** 2017-09-25

**Authors:** G Zhu, Q Qi, J J Havel, Z Li, Y Du, X Zhang, H Fu

**Affiliations:** 1 Department of Otolaryngology Head and Neck Surgery, Xiangya Hospital, Central South University, Changsha, China; 2 Department of Pharmacology, Emory University School of Medicine, Atlanta, GA, USA; 3 Department of Otolaryngology Head and Neck Surgery, The Second Xiangya Hospital, Central South University, Changsha, China

## Abstract

PRAS40 has been shown to have a crucial role in the repression of mammalian target of rapamycin (mTOR). Nonetheless, PRAS40 appears to have an oncogenic function in cancer cells. Whether PRAS40 mediates signaling independent of mTOR inhibition in cancer cells remains elusive. Here PRAS40 overexpression in lung adenocarcinoma and cutaneous melanoma was significantly correlated to worse prognosis. And we identified an unexpected role for PRAS40 in the regulation of nuclear factor (NF)-κB signaling. P65, a subunit of the NF-κB transcription factor complex, was confirmed to associate with PRAS40 by glutathione *S*-transferase co-precipitation. Importantly, we found that PRAS40 can enhance NF-κB transcriptional activity in a manner dependent upon PRAS40–P65 association. Furthermore, we found that a small p65-derived peptide can disrupt the PRAS40–P65 association and significantly decrease NF-κB transcriptional activity. These findings may help elucidate the pleiotropic functions of PRAS40 in cells and suggest a novel therapeutic strategy in cancer patients with high expression of PRAS40 and NF-κB.

## Introduction

PRAS40 (proline-rich AKT substrate of 40 kDa, encoded by the gene *AKT1S1*), was first identified as a 14-3-3-binding protein.^1^ PRAS40 is known to inhibit mammalian target of rapamycin C1 (mTORC1) activity by binding Raptor and competing away binding of the mTOR substrates 4E-BP1 and p70S6K.^2, 3^ PKB/AKT and mTOR have been demonstrated to phosphorylate PRAS40 residues Thr^246^ and Ser^183/212/221^, respectively, *in vitro* and *in vivo*.^1, 2, 4, 5^ Activation of mTOR and AKT increases phosphorylation of PRAS40 and causes it to dissociate from Raptor, which subsequently relieves the inhibitory constraint on mTORC1-mediated phosphorylation of 4E-BP1 and p70S6K.^6^ PRAS40-mediated inhibition of mTORC1 is well-documented; however, there are several reports indicating that PRAS40 may have additional functions in cancer cells. For example, PRAS40 is illustrated as an antiapoptotic gatekeeper in early formation of embryoid bodies (three-dimensional aggregates of pluripotent stem cells).^7^ Knock out of PRAS40 in mice significantly decreased, rather than increased, phosphorylation of p70S6k.^8^ In addition, downregulation of PRAS40 reduced the phosphorylation of AKT and mTORC1-regulated proteins in primary human skeletal muscle cells.^9^ Importantly, PRAS40 knockdown in murine tumor models reduce tumor growth and development in malignant melanoma and Ewing sarcoma.^10, 11^ Given the above evidence, we hypothesized that the function of PRAS40 may not be limited to mTORC1 inhibition. Here we found that high expression of PRAS40 mRNA indicated short survival time of patients with lung adenocarcinoma and cutaneous melanoma. Indeed, we previously showed that nuclear PRAS40 suppresses p53-dependent senescence via physical association with RPL11.^12^ And we demonstrate that PRAS40 can associate with p65 and modulate NF-κB transcriptional activity in this study.

It is well known that P65 dimerizes to form the core of the NF-κB complex.^13^ Our findings indicate that PRAS40 can promote NF-κB transcriptional activity through association with P65. Furthermore, a small peptide derived from P65 can disrupt the PRAS40–p65 association and reduce NF-κB transcriptional activity. These discoveries provide important insights into the pleiotropic functions of PRAS40 within cancer cells and reveal the PRAS40/P65 interaction interface as a potential site for therapeutic intervention.

## Results

### Elevated expression of PRAS40 predicts unfavorable survival in lung adenocarcinoma and cutaneous melanoma

**Figure 1:**
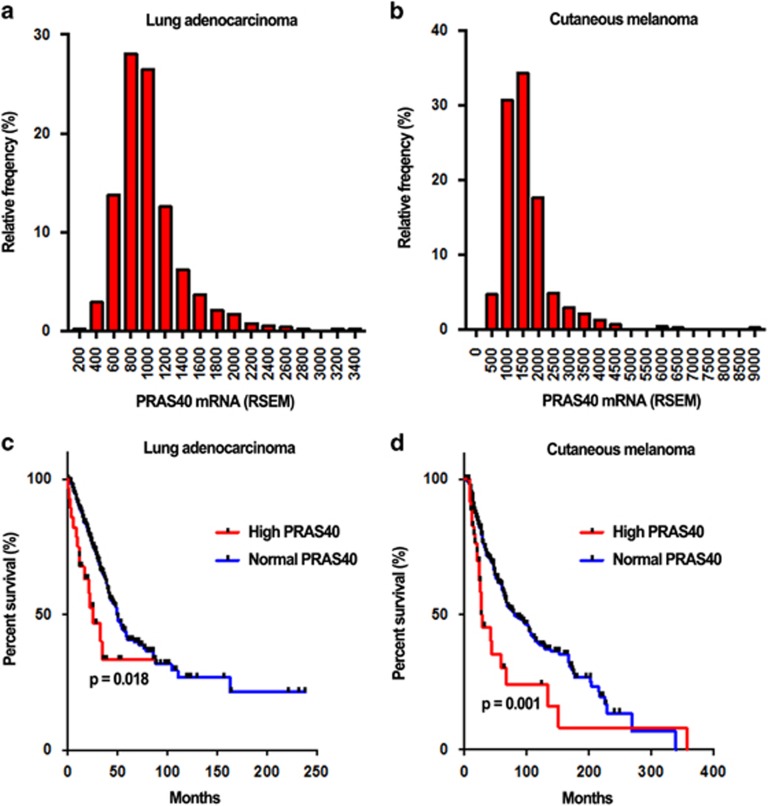
PRAS40 overexpression predicts adverse survival time of lung adenocarcinoma and cutaneous melanoma. (**a**, **b**) The distribution of PRAS40 mRNA expression in lung adenocarcinoma (**a**) and cutaneous melanoma (**b**), *x* axis, RSEM of PRAS40 mRNA; *y* axis, the relative frequency corresponding to RSEM in *x* axis. (**c**, **d**) Kaplan–Meier plots for survival analysis were created and compared between the PRAS40-High and PRAS40-Normal groups in lung adenocarcinoma (**c**) and cutaneous melanoma (**d**).

The clinical parameters, survival time and PRAS40 expression of 522 lung adenocarcinoma and 479 cutaneous melanoma patients were downloaded from cBioPortal (http://www.cbioportal.org/), which was generated from The Cancer Genome Atlas (TCGA) network. As shown in Figures 1a and b, PRAS40 was overexpressed in the top 28 (5.6%) and 36 (8.5%) expression values for lung adenocarcinoma and cutaneous melanoma, respectively.

Kaplan–Meier analysis implied that there was significant difference in survival time between patients with PRAS40-High and PRAS40-Normal expression (Figures 1c and d). The median survival time of 496 lung adenocarcinoma patients were only 25.5 months in the group of PRAS40-High expression but 49.3 months in the group of PRAS40-Normal expression. For 424 cutaneous melanoma patients, the median survival time were 27.2 months in PRAS40-High expression patients comparing to 80.6 months in PRAS40-Normal expression.

### PRAS40 associates with P65

**Figure 2:**
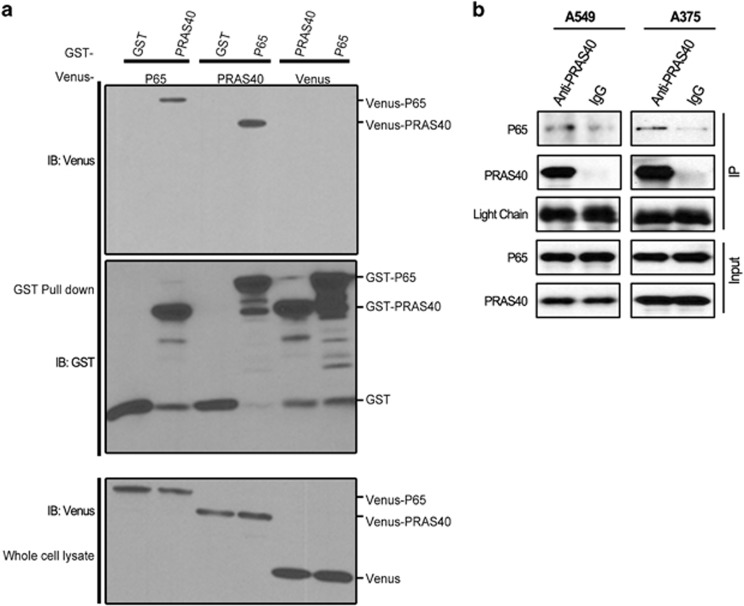
PRAS40 associates with P65. (**a**) Plasmids expressing GST-PRAS40, GST-only, Venus-p65 or Venus only were transfected as indicated. Glutathione beads were used to precipitate GST-tagged proteins. Eluates and input lysates were analyzed by western blotting. (**b**) Endogenous PRAS40 was co-immunoprecipitated with p65 in melanoma A375 and lung cancer A549 cells.

To examine the potential mechanism for a protumorigenic role of PRAS40, we carried out a protein interaction network-based studies and identified P65 as a PRAS40 partner. To provide orthogonal validation evidence for the putative PRAS40–p65 association, a glutathione *S*-transferase (GST) pull-down experiment was used. As shown in Figure 2a, overexpressed P65 was co-precipitated by GST-PRAS40 and *vice versa*. GST alone did not co-precipitate PRAS40, demonstrating that the PRAS40–p65 co-precipitation is not an artifact of non-specific GST binding. Furthermore, in A375 human melanoma and A549 lung cancer cells, interaction of PRAS40 and p65 was confirmed at endogenous levels by co-immunoprecipitation assay (Figure 2b).

### PRAS40 promotes transcriptional activity of NF-κB

**Figure 3:**
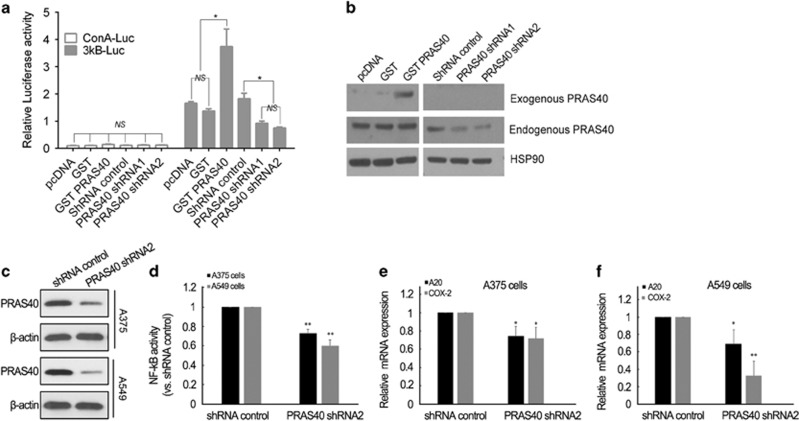
PRAS40 regulates NF-κB transcriptional activity. (**a**) Dual Luciferase Reporter assay of NF-κB transcriptional activity. ConA-Luc and 3κB-Luc was the Firefly plasmid for the control and reporter, respectively. Renilla was transfected for the endogenous control for the experiment. The ratio of Firefly to Renilla is presented on the *y* axis. The activity of 3κB-Luc in the cells expressing GST-PRAS40 was significantly enhanced relative to cells transfected with the GST control and pcDNA (*P*<0.001). Two PRAS40-targeted shRNAs significantly decreased the relative activity of 3κB-Luc compared with control shRNA (*P*<0.01). There were no significant differences of relative activity between the groups of ConA-Luc. NS=*P>*0.05, *P<0.05. (**b**) In order to determine the transfection efficiency, the cell lysates were analyzed by western blotting. HSP90 was used as a loading control. (**c**) Confirmation of PRAS40 knocking down in melanoma A375 and lung cancer A549 cells. (**d**) Knocking down of PRAS40 suppressed NF-κB p65 DNA-binding activity in both A375 and A549 cells. Data are expressed as mean±s.d. *P<0.05, **P<0.01. (**e**, **f**) Knocking down of PRAS40 downregulated A20 and COX-2 mRNA levels in A375 (**e**) and A549 (**f**) cells. Data are expressed as mean±s.d. *P<0.05, **P<0.01.

P65 is a subunit of the canonical NF-κB transcription factor heterodimer. We therefore analyzed whether PRAS40 could modulate NF-κB transcriptional activity. A dual luciferase reporter assay showed that overexpressed PRAS40 promoted NF-κB significantly (*P*<0.001, Figure 3a). To control for off-target effects, two different PRAS40 short hairpin RNAs (shRNAs) were used to deplete PRAS40. Both shRNAs reduced NF-κB transcriptional activity to a similar extent (both *P*<0.001, Figure 3a). The transfection efficiency was validated by western blotting of the same cell lysates used in the reporter assay (Figure 3b). To validate the induction of NF-κB activity by PRAS40 in cancer cells, we examined p65–DNA-binding activity and mRNA levels of A20 and COX-2, two target genes of NF-κB in cancer cells with normal expression and knocking down of PRAS40. As shown in Figures 3d–f, following knocking down of PRAS40 (Figure 3c), p65–DNA-binding activity as well as the expression levels of A20/COX-2 mRNA were significantly decreased, indicating the upregulation of NF- κB activity by PRAS40.

### PRAS40 residues 98-110 are sufficient for p65 association

**Figure 4:**
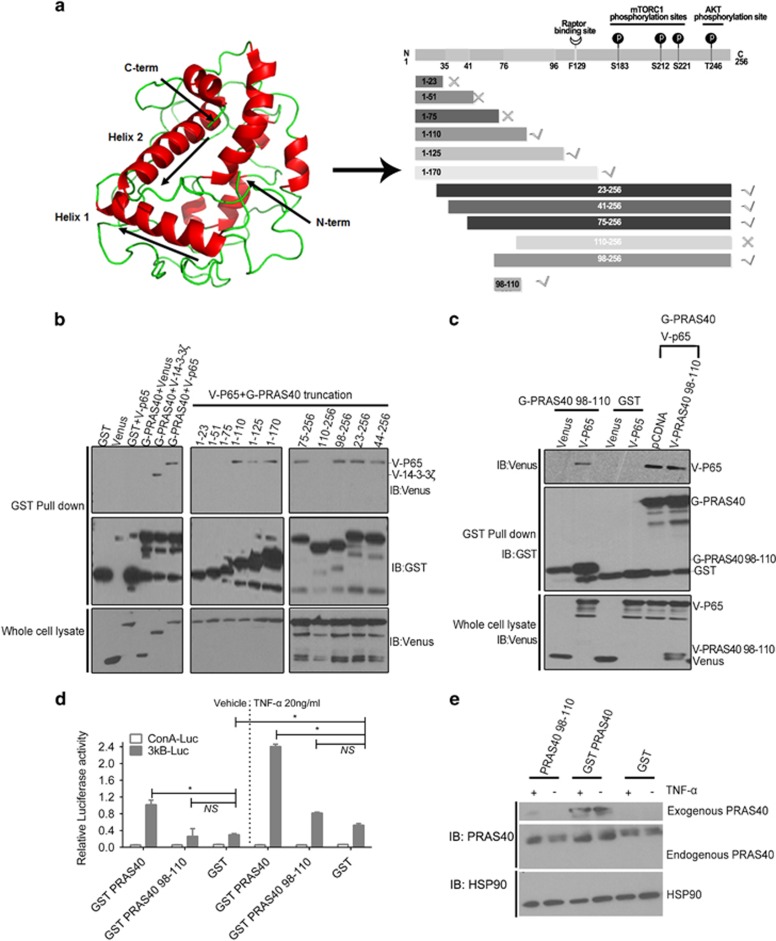
PRAS40 98-110 is sufficient but not required for PRAS40–P65 association. (**a**) The PRAS40 structure was predicted by the I-TASSER algorithm and visualized using the PyMOL software (Left). The truncations of PRAS40 were produced based on the predicted structure of PRAS40 (Right). ✓ means could interact with P65; × means could not interact with P65 according to the results of a GST co-precipitation assay (**b**, **c**). (**b**) Truncations of PRAS40 with GST tag protein were co-transfected withVenus-P65 (V-P65, P65 protein with the Venus-Flag tag). The GST, Venus and truncations of PRAS40 (1-23, 1-51,1-75 and 110-256) could not co-precipitate P65 (lanes 1–3). However, the full length of PRAS40 and the truncations, including 1-110, 1-125, 1-170, 75-256, 98-256, 23-256 and 44-256 of PRAS40, could co-precipitate P65. 14-3-3ζwas used as the positive control for PRAS40 binding in this experiment. (**c**) The small truncation 98-110 of PRAS40 could co-precipitate P65 (lane 2 vs lane 1). But it could not significantly affect the co-precipitation of P65 by full-length PRAS40 (lane 6 vs lane 5). Lanes 3 and 4 were negative controls. (**d**) The 98-100 truncation of PRAS40 did not significantly regulate NF-κB transcriptional activity with or without tumor necrosis factor-α (TNF-α) stimulation (20 ng/ml, 2 h). (**e**) The proteins in the 3κB-Luc group cells were blotted to validate the transfection.

After confirming the physical association of PRAS40 and p65, we next sought to identify the minimal region(s) of PRAS40 and p65 required for association. We first generated 11 truncated PRAS40 fragments and then tested which could interact with P65 via a GST pull-down experiment. Because a crystal structure of PRAS40 is unavailable, fragments of PRAS40 were designed according to a predicted homology-based 3D structural model generated by the I-TASSER algorithm^14^ (Figure 4a). As shown in Figure 4b, 110-256 of PRAS40 (lane 14) did not bind to P65, but 98-256 of PRAS40 (lane 15) did. Therefore, we hypothesized that residues 98-110 of PRAS40 may be a region required for PRAS40–p65 association. Co-precipitation results of other truncations, for example, 1-110, 1-125 and 1-175, are consistent with this hypothesis: all truncations including the 98-110 region could co-precipitate p65 (Figure 4b). To further evaluate this hypothesis, the PRAS40 98-110 fragment was expressed and found to co-precipitate p65 (Figure 4c, lanes 1–4). However, the PRAS40 98-110 fragment could not significantly disrupt co-precipitation of full-length PRAS40 and P65 (Figure 4c, lanes 5 and 6). Furthermore, PRAS40 98-110 did not regulate NF-κB transcriptional activity with or without tumor necrosis factor-α treatment (Figure 4d). Taken together, these results suggest that, while PRAS40 residues 98-110 are sufficient for p65 association, other regions of PRAS40 may also be involved.

### p65 residues 180-200 are sufficient for PRAS40 association

**Figure 5:**
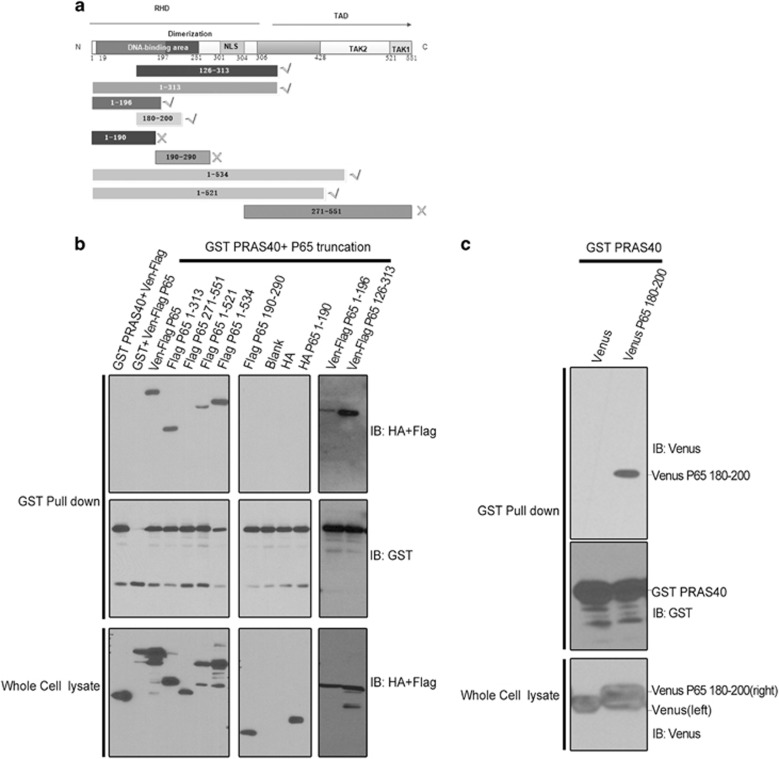
Residues 180-200 of p65 are sufficient for PRAS40 association. (**a**) Truncations of P65 were made based on its known structure and function. RHD represents Rel Homology Domain for the function of dimerization and TAD means transcription activation domain. ✓ means could interact with PRAS40; × means could not interact with PRAS40 according to the results of a GST co-precipitation assay (**b**, **c**). (**b**) Left blot: Full-length and truncations of P65 (1-313, 1-521, 1-534) could be co-precipitated by PRAS40. Middle blot: p65 truncations 190-290 and 1-190 of P65 could not be co-precipitated by PRAS40. Right blot: p65 truncations 1-196 and 126-313 could be co-precipitated by PRAS40. The remaining lanes were the negative controls. (**c**) A Venus-tagged p65 180–200 peptide could be co-precipitated by PRAS40.

**Figure 6:**
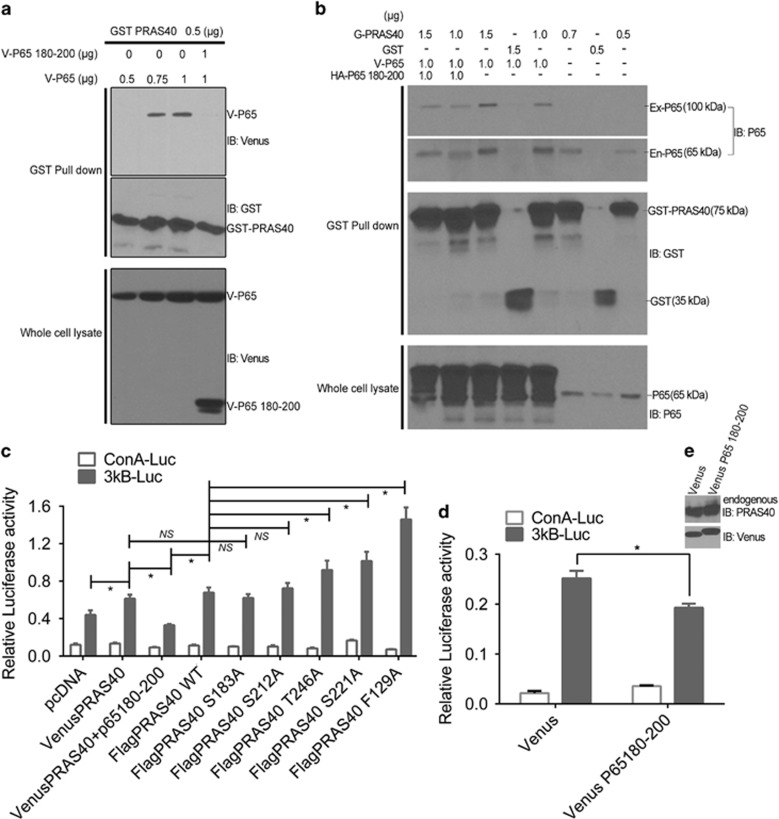
p65 180-200 peptide disrupts the PRAS40–P65 interaction and regulates NF-κB transcriptional activity. (**a**) The Venus-tagged p65 180-200 peptide could disrupt the PRAS40–P65 association (lane 4 vs lane 3). There is no significant difference between the cells co-transfected with GST PRAS40 DNA and 0.75 or 1 μg of Venus-P65 (lane 2 vs lane 3). (**b**) HA-tagged p65 180-200 could disrupt the PRAS40 association with exogenously expressed or endogenous p65 (lane 1 vs lane 3; lane 2 vs lane 5). The remaining blots served as negative controls. The blot of the Ex-/En-P65 shows the exogenous and endogenous P65 expression, respectively. (**c**) In all, 46.3% of NF-κB transcriptional activity in the 3κB-Luc group cells with PRAS40 overexpression was reduced by the 180-200 fragment of p65 (*P*<0.001). The mutants of PRAS40 increased the NF-κB transcription activity comparing to the pcDNA control group. There are no significant differences of relative activity between the groups of ConA-Luc. NS=*P>*0.05, *P<0.01. (**d**) The p65 180-200 peptide significantly reduced the basal NF-κB transcription activity in cells with endogenous PRAS40 expression. *P<0.01. (**e**) The 3κB-Luc group cells with Venus and Venus-P65 180-200 used in the above experiment (**d**) were utilized to validate the transfection efficiency. Flag was used as the referral control of transfection because flag tag was included in the Venus tag vector in this study.

We next determined which region of P65 is required for PRAS40 association. The structure of P65 has previously been solved. Truncations were generated based on p65 structure and function (Figure 5a). In this experiment, p65 residues 1-313, 1-521 and 1-534 and full-length p65 co-precipitated PRAS40, but 271-551 did not, indicating that residues 1-271 of P65 are important for the interaction (Figure 5b). The Rel homology domain (RHD) of P65 is known to mediate its heterodimerization. Based on the p65 3D structure, residue 190 is in the middle of a loop that connects two sides of the RHD and forms a pocket facilitating binding to other proteins (Figure 5). When the RHD was cut into 1-190 and 190-290, the pocket shape was deformed. Neither fragments1-190 nor 190-290 co-precipitated PRAS40 (Figure 5b), which led us to suspect that the region of p65 required for binding to PRAS40 may span residue 190. Taking together the above result that 1-271 is important for the interaction between PRAS40 and P65, we generated a plasmid encoding the 180-200 fragment of P65 and tested whether it could bind to PRAS40. As shown in Figure 4, p65 residues 180-200 were able to co-precipitate PRAS40 (Figure 5c). Furthermore, the p65 180-200 fragment could disrupt the association of GST-PRAS40 with exogenous or endogenous P65 (Figures 6a and b). To exclude the impact of the Venus tag on the disruption, we also used HA-tagged p65 180-200 and found that it produced the same results as the Venus-tagged version (Figure 6b, compare lanes 1 and 3 and lanes 2 and 5).

### Disrupting the PRAS40–p65 association reverses PRAS40-induced stimulation of NF-κB transcriptional activity

The p65 180-200 fragment provides a specific tool to study the function of PRAS40–P65 association because it can disrupt the interaction. As such, we tested whether the stimulatory effect of PRAS40 on NF-κB-mediated transcriptional activation is dependent on PRAS40–p65 association. The p65 180-200 fragment was able to decrease PRAS40-induced NF-κB-mediated transcriptional activity by 46.3% (Figure 6c, *P*<0.001), suggesting that PRAS40–p65 association is important for the stimulatory effect of PRAS40 on NF-κB transcriptional activity. Furthermore, the p65 180-200 fragment significantly reduced the NF-κB transcriptional activity induced by PRAS40 transfection (Figure 6d, *P*<0.001).

Both Akt and mTOR are involved in NF-κB activation,^15^ and PRAS40 residues T246 and F129 are AKT phosphorylation and Raptor-binding sites, respectively. Interestingly, the PRAS40 mutations (T246A and F129A) induced more NF-κB transcriptional activity than wild-type PRAS40 (Figure 6c).

## Discussion

Currently, the majority of research about PRAS40 molecular function is focused on the AKT-mTOR pathway. It is well established that PRAS40 can inhibit mTORC1 activity by binding to Raptor. AKT kinase can phosphorylate the T246 residue of PRAS40, causing subsequent dissociation of PRAS40 from mTORC1. Thereby AKT could relieve mTORC1 inhibition by phosphorylating PRAS40.^16^ As an inhibitor of mTOR, PRAS40 prevents mesangial cell and myocardium hypertrophy,^17, 18^ reduces H293E cell size and growth^2^ and improves insulin sensitivity.^9, 19^ Inactivating frameshift mutation of AKT1S1 has been detected in colorectal cancers.^20^ But depletion of PRAS40 has been found to decrease cell proliferation in nearly all the studies of PRAS40 in cancer cell models excluding colon cancer.^10, 11^ It remains unclear for the detailed mechanism of PRAS40 in cell prosurvival. Our previous work indicated that nuclear PRAS40 regulated cell senescence via the RPL11-HDM2-P53 nuclear stress response pathway.^12^ The current study showed that overexpression of PRAS40 correlated with poor prognosis of lung adenocarcinoma cancer and cutaneous melanoma patients, in agreement with animal and *in vitro* studies of malignant melanoma and Ewing sarcoma.^10, 11^ Excepting overexpression of PRAS40 in cancers, frameshift (fs) missense and splice mutations were found in various cancers, such as colorectal (P165fs), stomach (D238fs), cervical (P82Q), esophagus (E104K) and melanoma (D153_splice).^21^ We go on to demonstrate a novel physical association between PRAS40 and p65.

The p65–p50 heterodimer is a prototypical NF-κB transcription factor complex in mammalian cell.^22^ NF-κB, originally described as an immunoglobulin k light chain enhancer-binding protein,^23^ has been shown to enhance transcription of many other genes, including oncogenes and tumor suppressors, in a large-scale investigation.^24^ Inducible NF-κB transcriptional activity has a crucial role in immune system function and inflammation, as well as in oncogenesis by governing cell proliferation, apoptosis, differentiation and metastasis in response to dynamic environmental cues.^23^ Targeting NF-κB activity in cells may halt inflammation and slow tumor development. According to our results, PRAS40 forms a physical complex with p65 and promotes NF-κB transcriptional activity; however, the mechanistic details of this phenomenon remain to be elucidated. Our results indicate that overexpressed PRAS40 significantly promotes the transcriptional activity of NF-κB, while PRAS40 KD decreases NF-κB activity. Interestingly, both our results and those of a genome-wide small interfering RNA screen^25^ uncovered this novel molecular function of PRAS40 on NF-κB activity. The regulation of NF-κB activity by various stimuli such as cytokines or infection requires the degradation of IκB α, which unmasks and releases p65 from the cytoplasm into the nucleus.^15, 26^ However, our results showed that altered expression of PRAS40 did not impact phosphorylation of IκBα (Supplementary Figure S1), which may indicate that PRAS40 promotes the transcriptional activity of NF-κB by binding to P65 in the nucleus or through another non-canonical mechanism. Akt-mediated PRAS40 phosphorylation was reported to result in nuclear localization.^27^ Taken together with our results, this may suggest that Akt activates NF-κB transcriptional activity partly through phosphorylation of PRAS40.^28^ PRAS40 directly binds and inhibits mTORC1. This binding is dependent upon PRAS40 residue F129 and competes for phosphorylation of the primary mTORC1 substrates, 4E-BP1 and S6K1. The F129A PRAS40 mutation renders PRAS40 incapable of binding or inhibiting mTORC1.^3^ This study found that the PRAS40 F129A promotes NF-κB transcriptional activity to a greater extent than does wild-type PRAS40, suggesting that PRAS40–mTORC1 binding and PRAS40-mediated NF-κB activation may be mutually exclusive events.

Our results not only show the interaction of PRAS40 and p65 at overexpression system (Figure 2a) but also at endogenous level (Figure 2b) without any stimulation, indication that the binding constitutively exists in cancer. Moreover, knocking down of PRA40 decreases NF-κB transcriptional activity and suppresses transcriptions of A20 and COX-2, two target gene of NF-κB (Figure 3), suggesting the role of interaction of PRA40/p65 in cancer cell survival or growth. In order to further investigate whether the regulation of NF-κB transcriptional activity by PRAS40 depends on the specific association between PRAS40 and P65, the regions required for this interaction were narrowed via mapping several different truncations of P65 and PRAS40 protein. Several truncations derived from PRAS40 were generated according to its predicted structure. The overlap region of fragments derived from PRAS40, which could bind to P65, were amino acids 98-110. Therefore, we concluded that PRAS40 98-110 is involved in the PRAS40–P65 interaction. However, our GST pull down experiment showed that PRAS40 98-110 could bind to P65 but could not disrupt the PRAS40–P65 interaction. This may indicate that PRAS40 98-110 is sufficient but not necessarily required for PRAS40 binding with P65. There is no significant difference in endogenous NF-κB activity between the PRAS40 98-110 and transfection control groups with or without tumor necrosis factor-α stimulation (Figure 3d). Perhaps 98-110 is sufficient for binding, but other, likely weaker, PRAS40 contacts can cooperatively compensate for p65 binding in the context of 98-110 peptide overexpression. Therefore, we next focused on truncations derived from P65.

The study presented that PRAS40 could bind to all the truncations, including RHD of P65 (1-313 amino acids, RHD domain). RHD domain in P65 was demonstrated to be responsible for DNA binding and heterodimerization/homodimerization.^29, 30^ Many NF-κB co-activators are reported to moderate NF-κB transcriptional activity by interacting with the RHD domain,^31^ which may support our result that the RHD domain of P65 has a crucial role in the PRAS40–P65 interaction. Further, a small p65 peptide (P65 180-200, the 180th–200th amino acids from N terminal to C terminal) derived from the RHD domain of P65 was capable of interacting with PRAS40 and disrupting the association between PRAS40 and exogenously expressed or endogenous P65. Interestingly, according to the solved crystal structure of p65^32^ (http://www.rcsb.org), residues 180-200 of P65 form a pocket that may provide a dock for PRAS40 binding. Because the effect of P65 180-200 on other known RHD-binding partners remains unknown, PRAS40 may be the first known binding partner of this particular region of the p65 RHD. Notably, the putative PRAS40–p65-binding interface includes positively charged amino acids (K/R/H) on the P65 180-200 fragment (amino acids: SHPIFDNRAPNTAELKICRVN) and negatively charged amino acids (E/D) on the PRAS40 98-110 fragment (LAREDNEEDEDEP). As the binding sites of PRAS10/p65 and IκB/p65 are different, we cannot conclude that PRAS40 is a general mediator of canonical stimuli to NF-κB signaling. Furthermore, the NF-κB transcriptional activity induced by PRAS40 could be mainly offset by co-transfection with P65 180-200 in our dual luciferase reporter assay. Taken together with the known crucial role of NF-κB in lung cancer cells,^33^ our results indicate that the small fragment of P65 180-200 may be a promising clue to development of drugs targeting lung cancer with overexpression of PRAS40.

In summary, this work describes a novel protein interaction signaling node in lung cancer cells and demonstrates that PRAS40 can increase NF-κB transcriptional activity through physical association with P65. Future research will be needed to determine a detailed mechanistic relationship between PRAS40–P65 interaction and the oncogenic processes of lung cancer cells, and the discovery of a small truncation peptide (P65 180-200) in this study, capable of disrupting the PRAS40–p65 association, will facilitate future research in this area.

## Methods

### Plasmids and antibodies

All Flag-, GST-, Venus-(including Flag tag) and HA-tagged PRAS40 and p65 plasmids utilized in the study were generated by the Gateway cloning system (Invitrogen, Waltham, MA, USA). Human PRAS40 cDNA was obtained by PCR from a human tumor cDNA library. PRAS40 truncations and the fragment of P65 180-200 were generated via introduction of a stop codon during PCR amplification. P65 truncations were gifts from Dr Cunyun Wang except P65 180-200. All plasmids generated were confirmed by sequencing. shRNA for the control and PRAS40 plasmids were purchased from Open Biosystems (Lafayette, CO, USA, Vector control=RHS4346, PRAS40 shRNA1, 2=V3LHS_340055, V2LHS_138819) as we described previously.^12^ NF-κB luciferase reporter system: 3κB-Luc (Reporter) DNA and ConA-Luc (Control) DNA were kindly provided by Dr Neil D Perkins. Renilla luciferase DNA was purchased from Promega, Madison, WI, USA (pGL 4.74).

The following primary antibodies were used: Rabbit anti-PRAS40, Cell Signaling Technology, Boston, MA, USA, 2691S; Mouse anti-GAPDH, Millipore, Billerica, MA, USA; Rabbit anti-P65, Santa Cruz, sc-372; Rabbit anti-pan-14-3-3, Santa Cruz, Dallas, TX, USA, sc-629; Mouse anti-HSP90, Santa Cruz, sc-13119; Mouse anti-Flag, Sigma M2; Rabbit anti-GST, Santa Cruz, sc-459; Mouse anti-HA, Santa Cruz, sc-7392, Mouse anti-β-actin, Sigma, St Louis, MO, USA, A2228; Rabbit anti-IκB, Santa Cruz, sc-371; and Rabbit anti-phospho-IκB32/36, Cell Signaling Technology, 9246S.

### Cell culture and transfections

The HEK293T cell line was purchased from ATCC (Manassas, VA, USA) and maintained in Dulbecco’s modified Eagle’s medium (Cellgro, Manassas, VA, USA) media supplemented with 10% fetal bovine serum and 1 × Penicillin/Streptomycin Solution (Cellgro). Cells were incubated at 37 °C in humidified conditions with 5% CO_2_. FuGene HD (Roche, Basel, Switzerland) was used in a ratio of 3 μl to 1 μg DNA for cells according to the manufacturer’s instructions.

### GST pull-down and western blotting

As previously described, cells were lysed in CHAPS Buffer (40 mM HEPES pH 7.5, 120 mM NaCl, 1 mM EDTA, 0.3% CHAPS, 1:500 Sigma Phosphatase Inhibitor Cocktails 2 and 3 and Protease Inhibitor Cocktail). In all, 10% of the cell lysates were kept as the input. And 90% of the cell lysates were utilized as pull down and incubated with glutathione-conjugated beads (GE, Little Chalfont, UK) over night at 4 °C. These beads were washed three times with ice-cold CHAPS Buffer and then eluted by boiling 5 min in sodium dodecyl sulfate–polyacrylamide gel electrophoresis loading buffer. Both of the input and pull-down cell lysates were loaded for western blotting assay following the previous study.^12^

### Co-immunoprecipitation assay

Cell lysate was prepared with co-immunoprecipitation lysis buffer (50 mM Tris–HCl, pH 7.6, 150 mMNaCl, 1 mM EDTA, 1% (w/v) NP-40, 0.2 mM PMSF, 0.1 mM NaF). Cell lysate (1.5 mg total protein) was first incubated with 3 mg designated antibodies for overnight at 4 °C followed by incubation with protein A/G-Sepharose beads for 4 h at 4 °C. The beads were washed three times with ice-cold co-immunoprecipitation lysis buffer, resolved by sodium dodecyl sulfate–polyacrylamide gel electrophoresis and immunoblotted with antibodies against various proteins of interest.

### RNA extraction and real-time PCR assay

Total RNA was extracted using the miRNeasy Mini Kit (Qiagen, Hilden, Germany) according to the manufacturer’s instructions. RNA was reverse transcribed using the SuperScript III Reverse Transcriptase Kit (Invitrogen). PCR reactions were carried out in Eppendorf Mastercycler using the miCript SYBR Green Real-Time Kit (Bio-Rad, Hercules, CA, USA). The mRNA level of GAPDH (glyceraldehyde 3-phosphate dehydrogenase) was measured as an internal control. The PCR primers used are listed below: human A20 forward 5′-CTGCCCAGGAATGCTACAGATAC-3′, reverse 5′-GTGGAACAGCTCGGATTTCAG-3′ human COX-2 forward 5′-CACCCATGTCAAAACCGAGG-3′, reverse 5′-CCGGTGTTGAGCAGTTTTCTC-3′ and human GAPDH forward 5′-ATGTTCGTCATGGGTGTGAA-3′, reverse 5′-AGTTGTCATGGATGACCTTGG-3′.

### NF-κB transcriptional activity reporter assay and NF-κB activity assay

HEK293T cells with transfection of Renilla and NF-κB reporter plasmid or control plasmid as well as differential treatment relating to PRAS40 or P65 were lysed and detected in Dual-Glo Luciferase Assay System (Promega, E2920). Following the protocol of the manufacturers, Luciferase activity was measured by an Envision Multilabel plate reader (PerkinElmer, Houston, TX, USA). For NF-κB activity examination, it was analyzed using a NF-κB p65 Transcription Factor Assay Kit (Cayman Chem Co., Ann Arbor, MI, USA) according to the instructions of the provided protocol. (i) 10 μg of nuclear extract was added to the cell culture plate, which had already been incubated with 100 μl of p65 DNA sequence overnight at 4 °C). (ii) Each well was washed five times with 200 μl wash buffer. (iii) 100 μl diluted secondary antibody was added for 1 h. (iv) Each well was washed five times with 200 μl wash buffer. (v) 100 μl of developing solution per well was added for 45 min. (vi) 100 μl of stop solution per well was added for 5 min. (vii) Absorbance was measured at 450 nm.

### Statistical methods and software

PRAS40 expression and survival analysis in TCGA data RNAseq V2 RSEM-based expression and survival data were downloaded for lung adenocarcinoma (*n*=522) and cutaneous melanoma (*n*=479) from cBioPortal (http://www.cbioportal.org/index.do) (PMID: 23550210 and PMID: 22588877). The results are in whole based on data generated by the TCGA Research Network: http://cancergenome.nih.gov/. *Z*-scores for PRAS40 expression were calculated by cBioPortal based on the mean and s.d. of diploid tumor samples. *Z*>2 was used as the cutoff to define PRAS40 overexpression. This corresponded to the top 28 (5.6%) and 36 (8.5%) expression values for lung adenocarcinoma and cutaneous melanoma, respectively. For survival analysis, samples lacking tumor stage data were not included. Kaplan–Meier plots were created and patient survival was compared between the PRAS40-High and PRAS40-Normal groups by the log-rank test. The resulting *P*-values were adjusted to correct for patient age and tumor stage using multivariate regression. Statistical analyses were performed using the R Statistical Environment.

GraphPad Prism version 6.00 was used to perform statistical tests. Pooled data are reported as the means of independently repeated experiments ±s.d. Multiple comparisons were analyzed by Bonferroni’s Test embedded into the GraphPad Prism software (La Jolla, CA, USA). *P*-values are indicated in the figure legends.
