# Supplementary material for: PRAS40 promotes NF-κB transcriptional activity through association with p65
Source: Oncogenesis. 2017 Sep 25;6(9):e381–. doi: 10.1038/oncsis.2017.80 (PMC5623906; doi:10.1038/oncsis.2017.80)
Supplement: Supplementary Figure [file oncsis201780x1.docx]

**Supplementary Figure**

**Figure S1.** Over-expressed PRAS40 did not significantly affect the expression of p65 and the phosphorylation of Ser32/36 on IκB. β-actin was used as a loading control.
