# Supplementary figures and images for: PRAS40 promotes NF-κB transcriptional activity through association with p65
Source: Oncogenesis. 2017 Sep 25;6(9):e381–. doi: 10.1038/oncsis.2017.80 (PMC5623906; doi:10.1038/oncsis.2017.80)

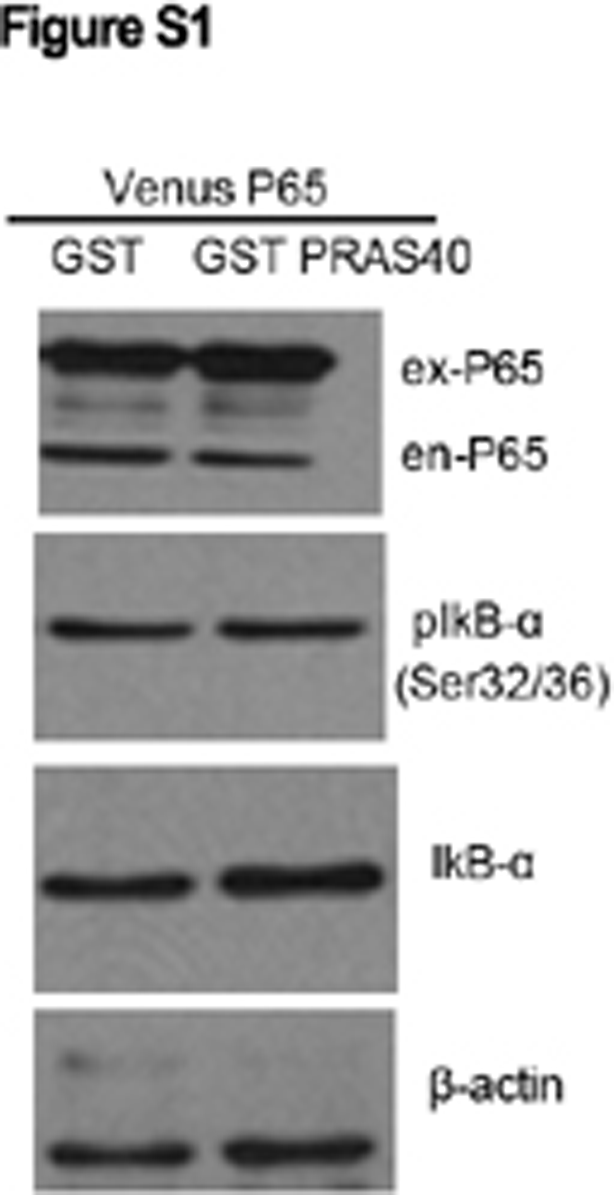

Supplement: Supplementary Figure [file oncsis201780x2.tif]
